# Risk Factors of Porcine Cysticercosis in the Eastern Cape Province, South Africa

**DOI:** 10.1371/journal.pone.0037718

**Published:** 2012-05-24

**Authors:** Rosina Claudia Krecek, Hamish Mohammed, Lynne Margaret Michael, Peter Mullineaux Schantz, Lulama Ntanjana, Liesl Morey, Stephen Rakem Werre, Arve Lee Willingham

**Affiliations:** 1 Department of Research, Ross University School of Veterinary Medicine (RUSVM), Basseterre, St. Kitts, West Indies; 2 Department of Zoology, University of Johannesburg, Auckland Park, South Africa; 3 University of Trinidad and Tobago, Arima, Trinidad; 4 Animal Health Division, Bayer Pty Ltd, Isando, South Africa; 5 Department of Global Health, Rollins School of Public Health, Emory University, Atlanta, Georgia, United States of America; 6 Department of Agriculture, Veterinary Services Eastern Cape Province, Bhisho, Eastern Cape, South Africa; 7 Biometry Unit, Agricultural Research Council, Silverton, Pretoria, South Africa; 8 Laboratory for Study Design and Statistical Analysis, Virginia-Maryland Regional College of Veterinary Medicine, Virginia Tech, Blacksburg, Virginia, United States of America; 9 Stewardship for Research on Infectious Diseases of Poverty (STE), UNICEF/UNDP/World Bank/WHO Special Programme for Research and Training in Tropical Diseases (TDR), Geneva, Switzerland; Mahidol Oxford Tropical Medicine Research Unit, Thailand

## Abstract

There is a high prevalence of *Taenia solium* taeniosis/cysticercosis in humans and pigs in the Eastern Cape Province (ECP) of South Africa. The objective of this study was to identify risk factors of porcine cysticercosis in select districts of the ECP. Data were collected in 2003 by interviewing 217 pig producers from the area. Blood samples were collected from 261 of their pigs, which were tested using two enzyme-linked immunosorbent assays (ELISA) for the presence of antibodies to cysticercosis. Frequencies of both owner- and pig-level characteristics were determined. For pig-level analysis, all bivariable and multivariable associations were determined using the surveylogistic procedure of the SAS/STAT® software to accommodate for the intraclass correlation that exists for clusters of pigs within one owner and for clusters of owners within a district. All tests for significance were performed at the α = 0.05 level, and adjusted odds ratios (aOR) and 95% confidence intervals (CI) were determined. Among the respondents, 48% of their households lacked a latrine, 98% slaughtered pigs at home, and 99% indicated that meat inspection services were not available. On bivariable analysis, there was a significant association between porcine infection and district (*p* = 0.003), breed (*p* = 0.041) and the absence of a latrine (*p* = 0.006). On multivariable analysis, the absence of a latrine was the only variable significantly associated with porcine infection (aOR = 1.89; 95% CI = 1.07, 3.35) (*p* = 0.028). The increased odds of porcine infection with households lacking a latrine contributes to our understanding of the transmission of this parasite in the ECP. Determining and addressing the risk factors for *T. solium* infection can potentially lower the very high prevalence in humans and pigs in this endemic area.

## Introduction

A high prevalence of *Taenia solium* taeniosis/cysticercosis is reported from some countries in Africa whereas limited or no information is available from others [1]–[2]. Cysticercois is a disease caused by infection with the larval stages of pork tapeworm, *T. solium*. [3]. Humans and pigs acquire cysticercosis by ingesting *T. solium* eggs. Neurocysticercosis (NCC) in humans occurs when cysts develop within the central nervous system. South Africa has the largest number of pigs (most being raised under commercial conditions) in southern Africa, and human and porcine cysticercosis has been recognized as a problem in the country for many decades [2], [4]–[7]. An extensive national abattoir study in 1937 reported a prevalence of 25% of porcine cysticercosis and an incidence of 10% in the Eastern Cape Province (ECP) of South Africa [1], [2]. The number of pigs continues to increase throughout southern Africa [3]–[7]. Approximately 25% of the pigs in South Africa are free-ranging and are owned by emerging pig producers (i.e. pig owners striving to increase production above subsistence) in resource-poor areas of the country. Additionally, projections for pork consumption in the developing regions of the world for the period 1993–2020 are anticipated to double (39 to 81 million tonnes) compared to a marginal increase in developed regions (38 to 41 million tonnes) [8].

The ECP reported high levels (28–50%) of human juvenile NCC (which occurs in children) and limited current data for porcine cysticercosis [5], [9]–[11]. In 2003, the prevalence of porcine cysticercosis was studied in these same districts by testing 261 pigs [10]. The diagnostic tests used in the study included lingual examination, two enzyme-linked immunosorbent tests (ELISA) and an enzyme immunoelectrotransfer (EITB) test [10]. Previously, when we employed a Bayesian approach to estimate the true prevalence of porcine cysticercosis in the ECP, we found the level to be very high [10]. In this study, using the same dataset, we determine risk factors of porcine cysticercosis, while accounting for the clustering effects of more than one pig belonging to the same owner and of more than one owner in a district.

## Materials and Methods

### Study design and population

This study was carried out from February to June 2003, in the six veterinary districts of the Alfred Nzo and Oliver R. Tambo Districts of the ECP. These districts included 124 villages and are served by animal health officers (AHO) and state veterinarians. The role of AHOs is to serve specific villages in a district. Therefore they contacted pig owners and invited them to participate in this study. The criteria for owners to be invited to participate in the study were that they were emerging pig producers, would be available during the days that the research team was scheduled to conduct the survey, and that the villages were accessible by road. All owners who agreed to participate were scheduled to meet on specific days with the research team. Details of the numbers of pigs and the village sampling are reported elsewhere [10].

### Household questionnaire

Data were collected from a standard hardcopy questionnaire that included 42 questions on household identification, information and respondent details, pig breed (South African hut breed, cross bred or other pure breed), pig management and husbandry, possible transmission factors (e.g. source of drinking water, absence of a latrine or toilet) and awareness of taeniosis/cysticercosis in humans and of cysticercosis in pigs. The questionnaire methodology followed [12]–[15] and I. Phiri (personal communication, 2003). The questionnaire underwent pilot testing in the field and then was administered to the pig owners during an interview by an AHO fluent in Xhosa (a local language) and concurrent with examination of the pigs. Due to the high level of illiteracy among villagers, informed verbal consent was obtained from pig owners by the AHOs.

### Statistical analysis

Data from the hardcopy questionnaires were entered into Microsoft Excel [16] and then exported to SAS/STAT ® software v9.2 [17] for analysis. Frequencies of both owner- and pig-level characteristics were determined. For pig-level analysis, all bivariable and multivariable associations were determined using the surveylogistic procedure.

Bivariable models were adjusted for clusters of pigs within owner, while the multivariable model was simultaneously adjusted for clusters of pigs within owner and clusters of owners within district. Porcine infection with cysticercosis was determined using two ELISA tests. B158/B60 Ag-ELISA [18] and HP10 Ag-ELISA [19]–[20]. The outcome for all analyses was porcine infection, defined as a positive result on either of the two ELISA tests. All variables with a *p*-value less than or equal to 0.10 on bivariable analysis were considered for inclusion in the final multivariable model, and tests for significant interaction between the terms in this model were performed. All tests for significance were performed at the α = 0.05 level, and both unadjusted and adjusted odds ratios (aOR) and 95% confidence intervals (CI) were determined.

### Ethical approval

The study protocol was approved by the National Department of Agriculture Directorate, South Africa and Animal Ethics Committee of Veterinary Services Eastern Cape Province, Department of Agriculture, ECP, South Africa, village leaders and pig owners. Collection of blood specimens from pigs was performed by a licensed veterinarian according to South African guidelines for animal care.

## Results

### Description of pig owners

A definitive account of the results for the true prevalence of porcine cysticercosis as well as operating characteristics of the laboratory tests used can be found in the previous papers [10], [21]. A total of 261 pigs, belonging to 217 owners (1∶1 owners to households), were sampled throughout the 21 study villages in the six veterinary districts. However, questionnaire data were available for only 256 pigs (98%); this was the sample size used for data analysis.

Of the pig owners interviewed, 73% were female; 50% were under 51 years of age; and 79% had less than or equal to standard 8 level of education. Only a few owners (12%) confined their pigs while the remainder (88%) utilized a free range system or semi-intensive system; 90% kept pigs for home consumption. Twenty percent of households obtained drinking water from taps, while 73% obtained their water from rivers, tanks, and boreholes. Forty-eight percent of households had no latrines, and their members defecated in the fields outside the homes.

The majority (93%) of households reported consumption of pork, 98% slaughtered pigs at home and almost all (99%) indicated that meat inspection services were not available. Most owners (74%) had observed cysts in pork, 78% did not know what the cysts were and 83% did not know how pigs acquired cysticercosis. The majority of respondents (69%) had heard of tapeworm infection in humans, though 74% did not know how to recognize a tapeworm if they were individually infected and 87% did not know how infection with a tapeworm occurred. The majority (86%) had heard of persons in their village complaining of epilepsy, 44% of headache and 62% of madness. Seventy-one percent reported that they did not know about the disease or how to treat, prevent or manage the disease.

### Risk factors and prevalence of infection

Overall, the prevalence (95% CI) of porcine infection was 57% (51%, 63%); 41% (35%, 47%) and 54% (48%, 60%) were positive by B158/B60 Ag-ELISA and HP10 Ag-ELISA, respectively. There was some variation between districts, with the prevalence of porcine infection ranging from 29% to 73% (Table 1). On bivariable analysis (Table 1), there was a significant association between infection and district (*p* = 0.003, type 3 wald statistic), breed (*p* = 0.041), and the absence of a latrine (*p* = 0.006). The multivariable model assessed included breed, absence of a latrine, and the interaction between breed and latrine as fixed effects; and owner and district as cluster level factors. In the final model, the absence of a latrine was the only variable significantly associated with infection (aOR = 1.89; 95% CI = 1.07, 3.35) (Table 2).

**Table 1:** Bivariable associations between owner/pig characteristics and cysticercosis^1^ infection in pigs from Eastern Cape Province (South Africa) (N = 256)^2^.

Risk factor	Category	Infected (n = 146)	Not infected (n = 110)	Crude odds ratio (95% CI)^4^	p-value
		n (%)^3^	n (%)^3^		
Veterinary district^5^	Umzimukulu	28 (58)	20 (42)	1.17 ( 0.53 , 2.59 )	0.691
	Maluti	17 (49)	18 (51)	0.79 ( 0.34 , 1.85 )	0.590
	Tsolo	32 (71)	13 (29)	2.06 ( 0.89 , 4.78 )	0.091
	Qumbu	29 (73)	11 (28)	2.21 ( 0.92 , 5.29 )	0.075
	Lusikisiki	9 (29)	22 (71)	0.34 ( 0.14 , 0.86 )	0.022
	Mt. Frere	31 (54)	26 (46)	Reference	
Breed^6^	Cross bred	18 (40)	27 (60)	0.42 ( 0.22 , 0.83 )	0.012
	Other	2 (67)	1 (33)	1.27 ( 0.08 , 20.97 )	0.868
	Hut pig	123 (61)	78 (39)	Reference	
Latrine^7^	Absent	82 (66)	42 (34)	2.08 ( 1.24 , 3.49 )	0.006
	Present	63 (48)	67 (52)	Reference	
Husbandry system^8^	Confined	23 (56)	18 (44)	0.95 ( 0.48 , 1.85 )	0.870
	Free-ranging/semi-intensive	123 (57)	91 (43)	Reference	
Measles given as a main problem with pig rearing^9^	Yes	57 (52)	53 (48)	0.67 ( 0.4 , 1.13 )	0.131
	No	88 (62)	55 (38)	Reference	
Source of drinking water^10^	Tap water	27 (54)	23 (46)	0.86 ( 0.45 , 1.63 )	0.635
	Natural sources or boreholes	118 (58)	86 (42)	Reference	
Consumption of pork at home^11^	Yes	135 (56)	104 (44)	Reference	0.500
	No	6 (67)	3 (33)	1.54 ( 0.44 , 5.42 )	
Meat inspected by a meat inspector^12^	Yes	0 (0)	0 (0)	§	-
	No	145 (57)	109 (43)	Reference	
Pigs slaughtered at home^13^	Yes	142 (57)	109 (43)	Reference	
	No	3 (100)	0 (0)	§	-

§ - not determined, due to cells with zero counts.

‵1 Determined using two enzyme-linked immunosorbent assays (B158/B60 Ag-ELISA [18] and HP10 Ag-ELISA [19]–[20]). Infection was defined as a positive result on either of these two ELISA tests.

2 Analyses were performed using the survey logistic procedure of the SAS/STAT® software to adjust for clusters of pigs within one owner.

3 (%) refers to row percentages.

4 CI = Confidence interval.

5 Type 3 wald statistic had a *p*-value of 0.003.

6 Type 3 wald statistic had a *p*-value of 0.041. Had missing values for n = 7. Other includes other pure bred breeds.

7 Had missing values for n = 2.

8 Had missing values for n = 1.

9 Had missing values for n = 3. Measles are cysts, from the larvae of *Taenia solium*, observed in pig meat.

10 Had missing values for n = 2.

11 Had missing values for n = 8.

12 Had missing values for n = 2.

13 Had missing values for n = 2.

Note: no significant associations (*p*≥0.10) were found between porcine infection and owner or household variables (sex, age, education, years of experience managing pigs, keeping pigs for home consumption), or with awareness questions (knowing what cysts in pork were, knowing how pigs acquired tapeworm, knowing how humans were infected with tapeworm or how to recognize infection, or had heard of persons in the village suffering with epilepsy, headache, or madness).

**Table 2:** Final multivariable model for the association between owner/pig characteristics and cysticercosis^1^ infection in pigs from Eastern Cape Province (South Africa) (N = 256)^2^.

Risk factor	Category	Adjusted odds ratio (95% CI)^3^	*p*-value
Breed^4^	Cross bred	0.54 (0.26, 1.12)	0.099
	Other	1.80 (0.10, 31.38)	0.687
	Hut pig	Reference	
Latrine^5^	Absent	1.89 (1.07, 3.35)	0.028
	Present	Reference	

1 Determined using two enzyme-linked immunosorbent assays (B158/B60 Ag-ELISA [18] and HP10 Ag-ELISA [19]–[20]). Infection was defined as a positive result on either of these two ELISA tests.

2 Analyses were performed using the surveylogistic procedure of the SAS/STAT® software to simultaneously adjust for clusters of pigs within one owner and for clusters of owners within district of residence.

3 CI = Confidence interval.

4 Had missing values for n = 7.

5 Had missing values n = 2. Other includes other pure bred breeds.

## Discussion

This is the first community-based survey in an emerging pig producing and endemic area in South Africa. The objective of this study was to identify risk factors for porcine cysticercosis. As mentioned earlier in this paper, the true prevalence of porcine cysticercosis in the ECP was previously estimated, using Bayesian analysis, as 64.6% [10]. However, apparent prevalence estimates by each of the two ELISAs were inadvertently reversed in Table 1 of that paper; B158/B60 Ag-ELISA should have read 40.6% and HP10 Ag-ELISA as 54.8%. Similarly, the sequence of column headings in Table 4 of the prevalence paper [10] should have been “Number of pigs examined”, “Lingual examination”, “HP10 Ag-ELISA”, “B158/B60 Ag-ELISA” and “EITB”. These errors affected the outcome of the Bayesian analysis by overestimating the true prevalence. We re-ran the model, and it yielded a true prevalence of 56.7% [21].

Both the Bayesian and frequentist methods suggest a very high prevalence of this disease, especially when compared to other prevalence studies, using Ag-ELISA, in Zambia (30–52%) and Tanzania (3–48%) [22]–[23]. Some limitations to be borne in mind with this study include the lack of accessibility by road to all villages which may have influenced the representativeness of the sample to the target population and, though well validated Ag-ELISA tests were used, there may have been possible underestimation of the overall prevalences. Sensitivities' estimates for the B158/B60 and the Ag-ELISA tests were 63.3% and 70.4%, respectively [21].

Households lacking a latrine (or toilet) were associated with an 89% increase in the odds of porcine infection, and this contributes to our understanding of the transmission of this parasite in this study area. Our study agrees with those carried out in Zambia and Tanzania where the prevalence of porcine cysticercosis was considerably higher in pigs reared in households lacking latrines [22], [23]. In our study this may be explained by the easy access to human feces for the pigs in these areas. Though latrines and toilets were directly observed in the vicinity of homes, they were often a distance of 20–50 m from the house or were not in working order (R.C. Krecek, personal observation). In contrast, in Cameroon there was no association of infection in pigs in households without a latrine when compared to those with a latrine [24]. Another study in Mexico showed that latrines were available in the majority of households (91%) and none of the 53 pigs studied showed cysts in the tongue or antibodies against *T. solium* in western blot assays [25].

It is essential to consider the results of the current study in the context of the socioeconomic characteristics of the study population. Of the nine provinces in South Africa, the ECP continues to be the most challenged in terms of infrastructure. For example, when 2007 provincial and national figures are compared, the ECP reported the highest proportion (48%) of households with no toilets (or latrines) as compared to the provincial and national figures of 25% and 9% , respectively. This province also has the lowest proportion of homes with access to piped water (70% vs 89%). In addition, unemployment is second highest with 25% as compared to the national average of 21%. Levels of unemployment, the proportion of households without toilets and homes with access to piped water have improved across the country, but little has changed in this province since 2003 [10]. In addition, efforts to improve sanitary conditions throughout other provinces have been successful but there has been very little change in the ECP. This highlights the setting in which the transmission of porcine cysticercosis currently occurs [26].

The presence of *T. solium* limits pork production and is a serious threat to public health in this province [9]–[10]. The current study reports, though the majority of pig owners observe tapeworm infections in humans, they have a very poor understanding of the transmission of this parasite between humans and pigs. There is no access to formal meat inspection or availability of information about transmission, treatment and prevention of these diseases. Though these were not identified as risk factors the statistical analysis reported very high percentages. This is coupled with the high levels of porcine infection and human juvenile cysticercosis throughout the Alfred Nzo and Oliver R. Tambo Districts of ECP [2], [4]–[6]. An overarching goal of the Cysticercosis Working Group of Eastern and Southern Africa is to identify appropriate recommendations for prevention and management in endemic areas of these countries [2], [5], [10], [11]. In addition to the need for improvement of infrastructure is the need to provide appropriate information to communities. Education in combination with other interventions has been shown to have the greatest impact on lowering levels of these diseases [27] with one of the earliest reported examples in a rural community in Mexico [25], [27]. A more recent example tested the efficacy of teaching methods in Kenyan farmers to prevent human epilepsy caused by neurocysticercosis [28]. This training enhanced knowledge acquisition and behavior change which are first steps to prevention of this disease. Understanding the risk factors which lead to *T. solium* infection together with applying educational interventions to increase awareness and knowledge of this disease can potentially lower the very high prevalence in humans and pigs in endemic areas such as the ECP.
